# Evaluation of a Novel Tool for Apical Plug Formation during Apexification of Immature Teeth

**DOI:** 10.3390/ijerph19095304

**Published:** 2022-04-27

**Authors:** Yasser Alsayed Tolibah, Line Droubi, Saleh Alkurdi, Mohammad Tamer Abbara, Nada Bshara, Thuraya Lazkani, Chaza Kouchaji, Ibrahim Ali Ahmad, Ziad D. Baghdadi

**Affiliations:** 1 Department of Pediatric Dentistry, College of Dentistry, Damascus University, Al-Mazzeh St., Damascus P.O. Box 3062, Syria; firedragoon1994@hotmail.com (Y.A.T.); droubiline@gmail.com (L.D.); salekh1889@gmail.com (S.A.); gmmn2012@gmail.com (N.B.); shazako@yahoo.com (C.K.); 2 Department of Restorative Dentistry and Endodontics, College of Dentistry, Damascus University, Al-Mazzeh St., Damascus P.O. Box 3062, Syria; tamerabbara@gmail.com (M.T.A.); dr.thuraya1979@gmail.com (T.L.); 3 Dentistry Department, Al-Wakra Hospital, Hamad Medical Corporation, Al-Wakra P.O. Box 82228, Qatar; ibrahimali79@yahoo.com; 4 Department of Preventive Dental Science Division of Pediatric Dentistry, Dr. Gerald Niznick College of Dentistry, University of Manitoba, Winnipeg, MB R3E 0W2, Canada; 5 Toddlers to Teens Dental and Concord Surgical Centre, Winnipeg, MB R2L 0Y5, Canada; 6 Greenwoods Dental and Surgical Centre, Winnipeg, MB R3C 0B1, Canada

**Keywords:** MTA, Biodentine, amalgam carrier, MAP system, modified cannula, apical plug, dye extraction

## Abstract

The purpose of this study was to compare the sealing ability and time required for the formation of Biodentine and mineral trioxide aggregation (MTA) apical plugs, using three different delivery methods: an amalgam carrier (AC), the Micro Apical Placement (MAP) System or a novel tool using a modified cannula (MC). Materials and Methods: A total of 60 uniformed molar roots were divided into three main groups, according to the technique of apical plug formation: AC, MAP, and MC. Each group was divided into two subgroups, according to the filling material used: MTA and Biodentine. A timer was used to calculate the required time for apical plug formation. After setting the filling materials, the apical microleakage of the formed plugs was quantified using the dye extraction method and spectrophotometry. The differences between the groups were analyzed using the one-way ANOVA and LSD post hoc tests. The significance level was set at 0.05. Results: No significant differences were reported in the time required to form the apical plugs in all groups (*p* > 0.05). However, the apical plugs formed by the AC method had significantly higher microleakage than those formed using the MAP and MC methods (*p* < 0.05). Conclusion: Within the limitations of this study, the sealing ability of the apical plugs formed by the MC method is comparable to the MAP method and better than the AC method.

## 1. Introduction

The dentin formation and root development processes stop when an immature tooth undergoes pulp necrosis, due to dental caries or traumatic injuries. In such cases, the canal walls remain thin, and the root apex remains open. Consequently, achieving an adequate apical stop during canal preparation or obturation in these teeth is impaired, and the risk of extrusion of irrigants, medicaments, and root canal fillings beyond the apex is increased [1,2].

Apexification is a commonly used technique to close the apex by artificially inducing the formation of a calcified barrier, to allow condensation of the root filling materials and improve the apical seal [3,4]. Historically, calcium hydroxide was the material of choice to induce a calcified barrier at the apex. However, calcium hydroxide use is associated with handling difficulties, a very long treatment time (with repeated changes of the material over the course of 5 to 20 months, to induce the formation of a calcific barrier), the possibility of tooth fractures, and incomplete calcification of the bridge [5]. 

The success of the apexification procedure depends on the ability to create a homogenous and well-condensed apical plug, and this was introduced as an alternative of apexification for the treatment of open apices. The material used in creating the apical plug plays a vital role in the success of the apexification procedure. Ideally, the used material should be biocompatible with the periradicular tissues, impervious to dissolution or breakdown by the tissue fluids after setting, and capable of being adapted as closely as possible to the dentinal walls. Several materials have been used to form apical plugs, and, nowadays, calcium silicate-based materials, such as mineral trioxide aggregate (MTA and Biodentine), are among the most used in apexification procedures to form apical plugs [3,6,7,8].

Proper delivery and condensation of the plug material to the apical third of the canal is another essential requirement for creating an apical barrier. Several methods have been described in the literature, including the following:

1. Introducing the material into the canal orifice using an amalgam carrier (AC) and plugging it to the apical third with handle pluggers [7,9], large-sized gutta-percha [9,10], or large-sized paper points [11].
2. Specialized carriers, such as the Micro Apical Placement (MAP) System, deliver the material to the apical third of the canal and condense it with handle pluggers [12,13,14].
3. Using modified Thermafil carriers to deliver and condense the material in the apical third [15].
4. Using prefabricated apical plugs [16].

The ideal method for the delivery and application of apical plug materials into the canal is still controversial. Furthermore, the availability and cost of specialized delivery systems may preclude their use in general dental clinics. Some authors suggest that the success of the apical plug technique could be influenced by the intracanal delivery technique [17].

Therefore, the study aimed to introduce a novel tool (modified cannula—MC) for the formation of apical plugs and to compare the performance of the MC method with two commonly used methods (AC and MAP), concerning the sealing ability of apical plugs of MTA and Biodentine and the time required to form them. 

## 2. Materials and Methods

This study was approved by the Ethics Committee of Damascus University, Faculty of Dentistry (ID: 1403-9-3-2020). This article reports the findings of an experimental, in vitro portion of the study. 

### 2.1. Sample Selection, Preparation, and Distribution

The study used 60 recently extracted, permanent human maxillary and mandibular molars with at least a single, straight root with one canal. The teeth included here were extracted for orthodontic or periodontal reasons. The excluding criteria were teeth with cracks, open apices, internal or external resorption, or previous endodontic treatment. The teeth were decoronated by a 0.3 diamond disc (Tizkavan, Tehran, Iran) to obtain 60 roots with a standard length of 13 mm. The sample size was set based on a study by Adel et al. [18]. 

Apical patency was established with a #10 K-file (Dentsply Sirona, Ballaigues, Switzerland) until it appeared from the apical foramen, and the working length was recorded. The root canals were instrumented with Fanta AF3 (Fanta Dental Materials Co., Ltd., Shanghai, China) and irrigated with 1.3% sodium hypochlorite solution using a 27G needle (Sybron Endo, Crop, Orange, CA, USA). The apical size of the canals was standardized by manual instrumentation to size #60 K-files (Dentsply Sirona, Ballaigues, Switzerland) using the balanced force technique. The canals were irrigated with normal saline, filled with 1 mL QMix (Dentsply Sirona, Ballaigues, Switzerland) for 1 min as final irrigation, and then the canals were dried with paper points (Gabadent, Guangdong, China).

Samples were randomly divided into three main groups, according to the technique of apical plug formation (AC, MAP, and MC). Each group was then divided into two subgroups (MTA and Biodentine). Samples were given numbers and were randomized using a randomization site. Therefore, six experimental groups (*n* = 10) were established (Table 1). Finally, the roots were embedded in a moist sponge to simulate the periapical resistance present in a clinical setting and facilitate the application of plug materials. 

The MTA and Biodentine were mixed according to the manufacturer’s instructions. Each gram of MTA (White ProRoot MTA, Dentsply, Tulsa, OK, USA) powder was mixed with 0.34 g distilled water for 30 s on a glass board using a metal spatula. Each capsule of Biodentine (Septodont, St Maur-des-Fossés, France) powder was poured with 5 drops of Biodentine pipette liquid, and then it was mixed with an amalgamator (zzlincker, Zhengzhou, China) at 4000 rotation/min. 

### 2.2. Amalgam Carrier Method

The plug material was loaded in a regular-amalgam carrier (2 mm diameter) (Medesy, Italy) and injected at the canal orifice as a big batch. Then, it was down packed into the middle third with a size 3 hand plugger (Dentsply, Tulsa, OK, USA), and then with a size 2 hand plugger into the apical third. The process was repeated until the apical plug placement was of 4 mm thickness.

### 2.3. MAP System Method

The MAP One system (Produits Dentaires S. A., Vevey, Switzerland) includes one NiTi tip with a 1.10 mm diameter. The plug material was loaded into the carrier, delivered into the apical third in small batches, and condensed vertically with a pre-fitted hand plugger (size 2) until a 4 mm apical plug was created.

### 2.4. Modified Cannula Method

Cannulas consist of a needle, catheter, wings, injection part cap, and Luer lock plug.

**Figure 1:**
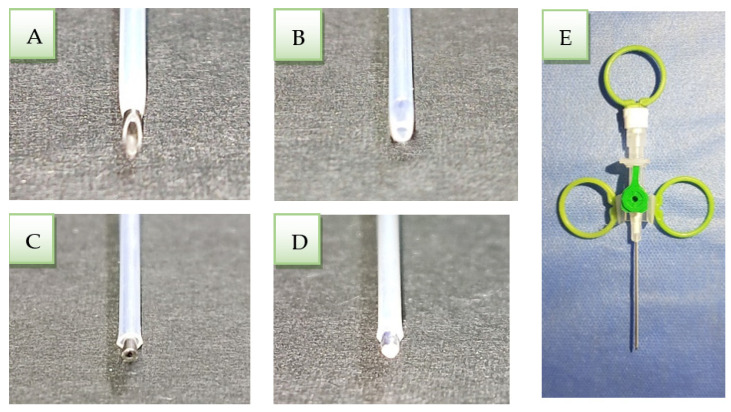
(**A**) The tip of the cannula before modifying. (**B**) The tip of the cannula after cutting the needle. (**C**) The clipped needle after cutting the plastic tube. (**D**) Sealing the clipped needle with silver weld. (**E**) The final shape of the modified cannula.

An 18-gauge size intravenous cannula (Figure 1A) was modified as follows:

1. A 2 mm cannula needle was cut with a diamond bur mounted on a high-speed handpiece perpendicularly to the vertical axis of the needle (Figure 1B).
2. The catheter was cut until 1 mm of the clipped needle appeared outside to push out the entire amount of cement inside the catheter when it reached the apical third of the canal (Figure 1C).
3. The clipped needle tip was closed with a silver weld to push the cement inside the catheter and condense the apical plug (Figure 1D).
4. Plastic rings were fixed to the cannula wings, and Luer lock plugs with silicon to make them easier to catch and use (Figure 1E).
5. The modified cannula was immersed in glutaraldehyde solution 2% for 24 h, then dried by air and kept in plastic wraps until use.

The modified cannula was loaded with the appropriate amount of plug material, delivered into the apical third, injected in small batches, and condensed vertically with the closed needle of the modified cannula. The process was repeated until the apical plug placement was of 4 mm thickness. All procedures were performed by a single specialist. 

**Figure 2:**
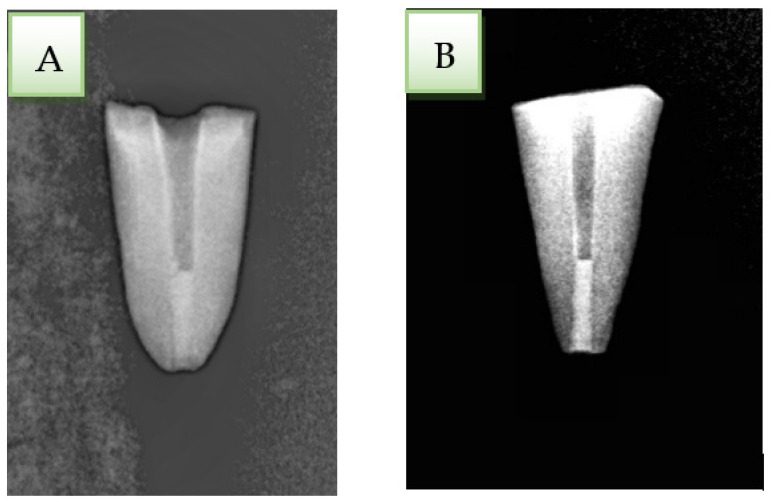
(**A**) Biodentine apical plug. (**B**) MTA apical plug.

After formation, a radiograph was taken of all apical plugs to ensure their quality (Figure 2). Finally, all samples were stored in a humid environment for 1 week to ensure that the materials were set.

A digital timer (Simex, Persiceto, BO, Italy) was used to calculate the time needed to load, place and condense both materials.

### 2.5. Marginal Leakage Testing

**Figure 3:**
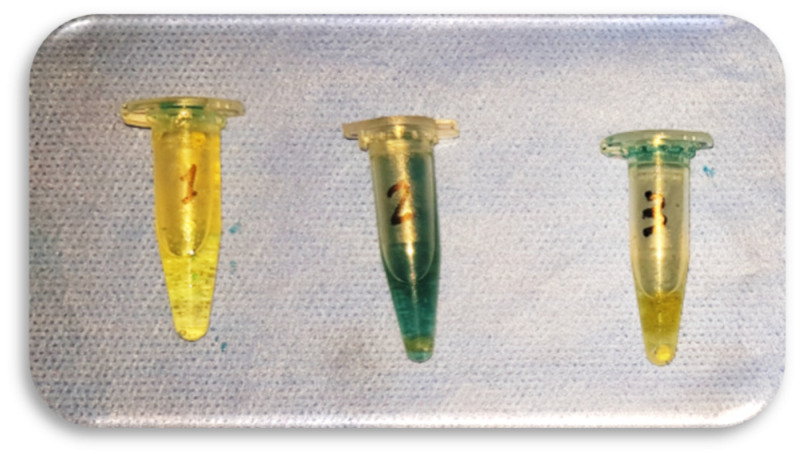
Dissolved roots with apical plug and pigment in nitric acid inside Eppendorf tubes.

Samples were coated with two coats of nail varnish, leaving 3 mm apically exposed. After the varnish was set, the samples were fixed on a wax board, where the apical third of the roots was immersed in a 2% methylene blue container and stored in an incubator (POL-EKO-APARATURA, Wodzislaw Slaski, Poland) at 37 °C for 72 h. Then, the samples were washed under running tap water to remove the dye traces, dried, and the nail varnish was removed with an ultrasonic scalpel tip. The sample was immersed in a test tube containing 1 mL of freshly prepared 65% nitric acid for 72 h to dissolve the root. Then, the obtained remaining solution was transferred to Eppendorf tubes (Seal-Rite^®^ Scientific, Inc., Ocala, FL, USA) (Figure 3) and centrifuged at 14,000 RPM in a centrifuge incubator (Andreas Hettich GmbH & Co. KG, Tuttlingen, Germany) for 5 min to separate debris from the extracted dye. The dye concentration in the supernatant solution was analyzed using a UV spectrophotometer (LW Scientific V325XS, Lab Essentials, Inc., Hicksville, NY, USA) at 550 nm, where the concentration of distilled water was taken as a blank.

### 2.6. Statistical Analysis

The data were analyzed by a statistician blinded to the type of apical plug material and the delivery method using the SPSS software (version 23, IBM SPSS Inc., Chicago, IL, USA). The normal distribution of data was evaluated using the Shapiro–Wilk test. Since the data were normally distributed, the mean times required to form the apical plug and the mean microleakage scores in all groups were compared using a one-way ANOVA test with the significance level set at *p* < 0.05.

## 3. Results

Table 2 summarizes the mean times required to form the apical plug in the study groups. The AC method was completed more quickly than the MAP and MC methods, regardless of the plug material type. However, the one-way ANOVA test showed no significant differences (*p* = 0.12).

**Figure 4:**
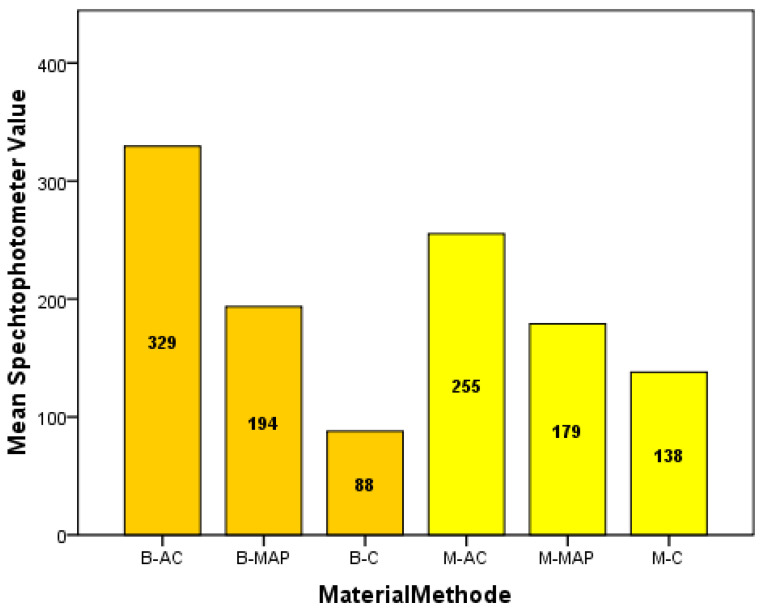
Mean spectrophotometer values.

Table 3 and Figure 4 summarize the mean apical leakage scores for the study groups. As the one-way ANOVA test showed a significant difference (*p* = 0.004) between the different groups, an LSD post hoc test was used for pairwise comparisons. The groups that used the AC methods (group 1: MTA-AC and group 2: Biodentine-AC) reported the highest apical leakage scores and significantly differed from the remaining groups.

## 4. Discussion

Calcium silicate-based materials are used in apical plugs. The apexification therapeutic approach was first introduced with the advent of MTA and, recently, Biodentine [19]. Biodentine maintains the preferable properties of MTA, and is designed to overcome the drawbacks of MTA, including tooth discoloration, poor handling characteristics, and long setting time, where Biodentine requires 12 min to complete its hardening [20]. The use of MTA and Biodentine in apexification procedures is considered as an alternative to the regenerative endodontic therapy and endodontic surgery options.

According to previous studies, many tools have been used to transfer calcium silicate cement into the root canal and plug it to form an apical plug. However, they may not be available in the dental clinic, due to their high price or the difficulty obtaining them, so the main idea of this research is to present a new tool (MC) for forming apical plugs and compare the sealing ability of the formed plugs with that formed with AC or MS.

Based on the commercially available MTA carriers in their various forms and high prices, it was found that intravenous cannulas are readily available tools that are cheap, disposable, and have a structure that is somewhat similar to the MTA carriers, with a reasonable price, where it is possible to make a simple adjustment to the cannula and sterilize it to use as calcium silicate-based cement carriers in the apical third of wide canals.

The plastic tube of the catheter is made of flexible reinforced plastic that remains protected, without bending, when the MC is inserted into the apical third of the canal, and its closed needle plays a plugger role when it condenses the apical plug.

This study included 60 intact straight roots of permanent molars that were extracted for orthodontic or periodontal reasons, and after cutting and preparing the roots, they had a uniform length and apex size.

The QMix irrigant has good smear layer removal ability [21], and it does not hamper the root dentin microhardness compared with conventional irrigants [22]. Moreover, the QMix irrigant does not affect the bond strength of MTA and Biodentine to the dentinal root surface [23,24], so it can be safely used as a final irrigate in the treatment of immature root canals before calcium silicate-based cement application as apical plugs.

Clinical studies on apical leakage and sealing continue to focus on intention because clinical failures still occur, despite the advances in endodontics. Most failures are probably attributed to the proliferation of bacteria that remain after chemical–mechanical preparation and cause apical lesions [25].

Different methods were suggested to evaluate the sealing ability of the apical plug, such as dye penetration, fluid filtration, glucose penetration, bacterial leakage, and dye extraction [25,26].

The dye extraction method has reliable results in microleakage studies [26], where all the dye that has escaped through the apex is recovered by dissolving it in acid, and it quantitatively measures the optical density of the solution using a spectrophotometer. Thus, it provides accurate numerical results for leakage, and this method has been used in some recent studies about the microleakage of apical plugs and root end materials [18,27,28].

A tight apical seal of the root canal system is an essential factor in the success of endodontic treatment [29]. Both MTA and Biodentine have a similarly good sealing ability when used as an apical plug [30], and the results of this research showed that regardless of the techniques used in the formation of the apical plug, the sealing ability of MTA and Biodentine was similar.

The results of this study showed that there are differences in the microleakage of apical plugs when forming with different techniques. When forming apical plugs using handle pluggers, they transfer the compressed calcium silicate cement mass directly to the apical third, with minimal compacting, which reduces the fragmentation and deformation of the total mass of the material, which keeps it coherent and free of voids and gaps. The clinical work that used this technique demonstrates its value when working with children [14,31] in clinical settings. Baghdadi previously evaluated some new dental materials using in vitro studies, including a microleakage method using a dye, but he emphasized that the ultimate test of dental materials is not their performance in the laboratory, but their performance in the clinical environment [32,33].

The groups that showed the most microleakage around the apical plugs were the AC, whether MTA or Biodentine. The reason for this may be that the material does not reach the apical third directly, and the compressed calcium silicate cement mass emerging from the amalgam carrier may be subjected to deformation, fragmentation, and void and gab formation as it rubs against the rough surface of the dentin while plugging the material from the orifice to the apical third.

The main limitation of this study is that its results cannot be compared with other in vitro studies results because this in vitro study is the first that studied the effect of tools that form apical plugs on its sealing ability; more clinical studies about this novel tool, to assess its quality, are warranted.

## 5. Conclusions

Within the current study’s limitations, the material delivery system affects the sealing ability of the apical plugs. The MC delivery method is comparable to the MAP method and better than the AC method.

## Figures and Tables

**Table 1:** Experimental groups.

	Group (*n* = 10)	Plug Material	Delivery Method
1	(MTA-AC)	MTA	Amalgam carrier
2	(MTA-MAP)	MTA	MAP System
3	(MTA-MC)	MTA	Modified cannula
4	(Biodentine-AC)	Biodentine	Amalgam carrier
5	(Biodentine-MAP)	Biodentine	MAP System
6	(Biodentine-MC)	Biodentine	Modified cannula

**Table 2:** Mean and range of apical plug formation time in seconds.

	Group (*n* = 10)	Mean ± SD	Range
1	(MTA-AC)	444.7 ± 155.2 (7.4 ± 2.5 min)	260–731
2	(MTA-MAP)	484.0 ± 176.5 (8.0 ± 2.9 min)	143–689
3	(MTA-MC)	516.3 ± 138.8 (8.6 ± 2.3 min)	272–644
4	(Biodentine-AC)	364.9 ± 119.6 (6.0 ± 1.9 min)	182–570
5	(Biodentine-MAP)	438.1 ± 145.1 (7.3 ± 2.4 min)	271–697
6	(Biodentine-MC)	552.5 ± 178.9 (9.2 ± 2.9 min)	261–829

**Table 3:** Mean and range of apical microleakage scores.

	Group (*n* = 10)	Mean ± SD	Range
1	(MTA-AC)	255.2 ± 174.0	45–482
2	(MTA-MAP)	179.0 ± 146.1	43–470
3	(MTA-MC)	138.0 ± 130.9	0–381
4	(Biodentine-AC)	329.4 ± 145.9	83–284
5	(Biodentine-MAP)	193.5 ± 128.9	42–426
6	(Biodentine-MC)	87.90 ± 73.3	22–237

## Data Availability

Deidentified data are available upon written request to the corresponding author.
